# Unicortical fixation does not compromise bony union in the Latarjet procedure

**DOI:** 10.1016/j.jseint.2022.04.007

**Published:** 2022-05-13

**Authors:** Shivan S. Jassim, Jeevaka Amaranath, David McD. Taylor, Sarah Ann Warby, Gregory Hoy

**Affiliations:** a Melbourne Orthopaedic Group, Melbourne, VIC, Australia; b Department of Medicine, University of Melbourne, Melbourne, VIC, Australia; c The Austin Hospital, Melbourne, VIC, Australia; d Melbourne Shoulder Group, Melbourne, VIC, Australia; e La Trobe University, Department of Rehabilitation, Nutrition and Sport, Corner of Kingsbury Drive and Plenty Road Bundoora, Melbourne, VIC, Australia; f Monash University, Department of Surgery, Melbourne, VIC, Australia

**Keywords:** Shoulder, Bicortical, Unicortical, Instability, Latarjet, Union

## Abstract

**Background:**

Fixation of the coracoid during the Latarjet procedure can be performed with either unicortical or bicortical fixation. There is no clear evidence that the number of cortices fixed affects graft union, but in vitro studies suggest bicortical fixation is desirable. The primary aim of the study was to retrospectively review the coracoid union rates in our Latarjet cohort who have undergone either unicortical or bicortical graft fixation.

**Methods:**

A retrospective review of Latarjet patients receiving bicortical or unicortical fixation was performed. The rate of coracoid graft union was assessed via radiographs and computed tomography scans at minimum 8 weeks postoperatively. Primary analysis for graft union was performed with Chi-squared and Fisher’s exact tests.

**Results:**

A total of 184 patients were enrolled (82 bicortical, 102 unicortical) with 20 patients lost to follow-up. There was no significant difference between union rates of bicortical and unicortical groups (union rate: 94% bicortical, 98% unicortical, *P* = .25). There were no significant differences in rate of instability recurrence (*P* = .5) or other postoperative complications (*P* = .83) between the groups.

**Discussion:**

At a minimum follow-up of 8 weeks, bicortical fixation was not shown to have a higher rate of union than unicortical fixation. Performing unicortical fixation is an acceptable practice without compromising bony graft union.

Traumatic anterior glenohumeral joint (GHJ) instability is reported as the most common type of shoulder instability, accounting for between 8 and 56 dislocations per 100,000 people per year.^36^^,^^42^ Due to the high rate of dislocation recurrence after a traumatic GHJ instability event, the recommended treatment option is commonly surgical stabilization, especially in the young sporting population.^13^^,^^23^ Surgical stabilization procedures broadly involve soft tissue (eg, arthroscopic Bankart repair) or bony augmentation procedures (eg, Latarjet procedure). Arthroscopic Bankart repair has been shown to be successful in reducing recurrent dislocations^32^; however, failure rates for this procedure have been reported between 8% and 23%.^14^^,^^35^ In cases of recurrent instability following primary stabilization, revision with an open stabilization in the form of the Latarjet procedure is often the next surgical option, especially in cases of significant glenoid bone loss.^11^^,^^17^^,^^22^ The Latarjet has also been performed successfully as a primary procedure when the consequences of stabilization failure and recurrence are too great, such as athletes playing semiprofessional or professional contact sport.^1^^,^^8^^,^^17^^,^^18^^,^^22^ The risks of higher intraoperative and postoperative complications (other than recurrence) with the Latarjet than with a capsulolabral repair^40^ and the possibility of early glenohumeral degenerative change^8^^,^^16^ need to be weighed up against the benefit of a low rate of recurrence in the context of the individual patient presentation and functional goals.^29^

In 1954, Latarjet described his procedure of transferring the coracoid process to the anterior margin of the glenoid.^21^ Its stabilizing mechanism was thought to be multimodal with increase of the glenoid surface area, the sling effect of the conjoint tendon, and repair of the capsulolabral complex to the bone block.^5^ There are different modifications to this technique, such as the position and alignment of the transferred coracoid (classical vs. congruent arc), repair of the capsulolabral structures, and the method of bone block fixation.^38^

Fixation of the coracoid graft to the glenoid can be performed with screws in either unicortical or bicortical configuration. Currently, there is controversary regarding which method to employ.^39^ Unicortical fixation may avoid soft-tissue irritation from prominent hardware^2^; however, biomechanical studies have demonstrated that pull-out strength of unicortical fixation is less than that of bicortical fixation.^34^^,^^39^ Reported limitations of the bicortical screw fixation include complications associated with protruding posterior glenoid screws, such as soft-tissue irritation or suprascapular nerve injury.^20^^,^^25^ Symptoms of screw irritation typically include anterior shoulder pain plus or minus weakness of shoulder movement.^9^ Due to the greater pull-out strength and a reduced risk of complications (such as pseudarthrosis of the coracoid process),^4^ recent recommendations have supported the use of bicortical graft fixation.^28^^,^^39^

Nonunion of the bone graft is a well-reported and clinically significant complication following the Latarjet procedure.^39^ A recent systematic review^8^ reported an estimated nonunion rate between 3.4% and 5.9% after Latarjet procedure; however, the screw configuration type was not specified. Currently, there is no clear in vivo evidence regarding the association between graft nonunion and the number of cortices fixed (unicortical or bicortical) in the Latarjet procedure.

In our practice, we perform the Latarjet procedure for a number of indications. These include significant glenoid bone loss (typically above 20%), revision from a failed soft-tissue repair, and as a primary procedure in contact sports athletes whose main aim is to reduce the risk of recurrent dislocation. Due to the strengths mentioned above, we have recently transitioned from using bicortical fixation to unicortical fixation of the coracoid in our Latarjet patients and have not noticed a demonstrable difference in the symptomatic or radiological nonunion rate of the graft.

Therefore, the primary aim of the study was to retrospectively review the coracoid union rates in our Latarjet patient cohort who have undergone either unicortical or bicortical coracoid graft fixation. The secondary aim was to report on patient-reported outcome measures (PROMs) and complications after the Latarjet procedure in both groups. We hypothesized that there will be no difference in union rates between patients undergoing unicortical vs. bicortical fixation of the glenoid.

## Materials and method

### Patient selection

This retrospective cohort study was performed at Melbourne Orthopaedic Group (33 The Avenue Windsor, 3181, Melbourne, Victoria, Australia) between March 2015 and May 2021. Ethical approval for this single-surgeon comparison of 2 Latarjet patient cohorts (unicortical and bicortical) was obtained (Ramsay Health Care NSW|VIC HREC 2019-LNR-024) as was written consent from all participants.

In April 2017, the principal surgeon (G.H.) changed their fixation method from bicortical fixation to unicortical fixation due to a change in screw design of the fixation device (DePuy MiTek, Raynham, MA, USA). Therefore, to obtain a near-equal sample size of each Latarjet group, the practice database was examined for all skeletally mature patients having undergone a bone block procedure performed between January 2015 and September 2019. We excluded other bony block procedures (such as an Eden-Hybinette procedure) and participants with incomplete operative information. The cohort was then divided into 2 groups based on the use of the older screw system (bicortical fixation group) before April 2017 and the new Synthes-Mitek screw system with sharper screw ends (unicortical fixation group) after April 2017.

The participant’s clinical notes were examined for coracoid graft fixation type, procedure type (eg, revision of a previous arthroscopic capsulolabral reconstruction or primary Latarjet), additional procedures performed, and any history of complications including recurrence of instability.

### Surgical technique

After informed consent was taken, the patient received an interscalene nerve block and a prophylactic dose of antibiotics. The patient was given a general anesthesia and placed into a semisupine position with the head over a neurosurgery head rest and the arm on an arm table.

A deltopectoral incision was made from the axillary fold to just proximal to the coracoid process. The cephalic vein was protected and taken laterally with tributary veins coagulated with diathermy. The coracoid process was exposed, and the conjoint tendon was dissected out, with the pectoralis minor tendon taken off the medial side of the coracoid process proximally. The clavipectoral fascia was débrided lateral to the conjoint tendon to expose the coracoid. A right-angled oscillating saw was used to remove 2 cm of the coracoid process with bone wax applied to the coracoid remnant. The coracoid was predrilled, and top hat washers were inserted. The undersurface of the coracoid was decorticated to bleeding bone and trimmed to the final shape to match the anterior glenoid bone bed similarly débrided to bleeding bone. Following identification of the musculocutaneous nerve, the conjoint tendon and bone block were tucked inferiorly while dissection into the joint was performed. A transverse subscapularis split was made with a transverse capsulotomy in the same plane, and a Fukuda retractor was placed over the humeral head and glenoid retractor onto the glenoid neck. The capsulolabral tissue was divided at the glenoid margin, and flaps reflected. Intra-articular findings were noted, the anterior glenoid bone was prepared by roughening the surface with a saw, and any detached segment of glenoid bone was removed prior to application of the coracoid bone block. A guide was used to position the coracoid with long guide wires passed across the glenoid and overdrilled with a cannulated drill. Depth of the titanium cannulated screw placement was determined initially with a length-marked drill and confirmed with a depth gauge through the posterior cortex.

For unicortical fixation, the screws were measured, and 4 mm removed from the measured distance. The smallest screw length was 28 mm, and the largest was 40 mm. The screws were inserted into each hole with good fixation of the coracoid bone block and seated with compression across the top hat washers. The newer Synthes designed screws had self-tapping sharp tips that were hypothesised to be of less risk to the posterior neurovascular structures if used with a unicortical fixation method. Using a double-loaded anchor, the labrum was reattached between the 2 screws, allowing a strong repair to the glenoid edge. The capsule was closed adjacent to the transferred bone block with pressure on the humeral head to assist tensioning, and retractors were removed. The subscapularis split was partially closed with no. 1 Vicryl sutures in the lateral half of the tenotomy only, in order to allow impingement-free internal rotation. The arm was taken through a range of motion to assess for stability and any impingement. A drain was inserted into the wound, and it was closed in layers with absorbable sutures. After a protective dressing was applied, the arm was placed in a sling with body band. Postoperative review by the surgeon occurred at 2, 8, and 16 weeks. A shoulder radiograph was performed at 8- and 16-week follow-ups to assess for union of the bone block as per standard practice.^8^^,^^15^ If there were any clinical or radiological concerns, a computed tomography (CT) was organized. Follow-up was continued until union had been radiographically confirmed and the patient reported an absence of symptoms (if symptoms were present).

Under the supervision of a physiotherapist, rehabilitation consisted of early limited motion from 2 weeks postoperatively, followed by active assisted to active range of motion from 4 weeks. Strengthening was initiated at 8 weeks after graft union was confirmed. Discharge from rehabilitation occurred when the patient had near to full range and full strength and did not have shoulder-related symptoms.

### Outcome measures

#### Radiographic analysis

Shoulder radiographs were performed at approximately 8 weeks postoperatively although not prior to the 8-week time point.^8^ Two senior orthopedic surgeons (S.A.W., J.A.) who were blinded to the method of fixation independently reviewed the radiographs for bony union by looking for cortical continuity between the bone block and glenoid on lateral and axial shoulder views. On x-ray, union was determined as cortical continuity of at least 3 of the 4 cortices in these orthogonal radiographs. On the CT scan using 2-mm interval slices, consecutive cortices between the graft and recipient bone were assessed as described by Makihara et al.^24^ At least 50% of traversing trabeculae throughout all axial CT slices was considered graft union.^34^ If union was not easily determined by plain x-ray or if there were any clinical concerns, subsequent CT scans were arranged.

The kappa statistic was employed to evaluate the intrareliability and interreliability of the 2 reviewers. Each independently reviewed the same random sample of 50 patients on 2 occasions, at least 1 week apart. The intrareliability of reviewers 1 and 2 were 1.0 (perfect agreement) and 0.66 (substantial agreement), respectively. The interrater reliability of the 2 reviewers was 0.66 (substantial agreement).

#### Recurrence of instability and postoperative complications

For included participants, the incidence of a recurrence of GHJ instability as well as any postoperative complications was extracted and recorded by 2 of the authors (S.J., J.A.). A recurrence of instability was defined as a patient-perceived moment in time of either full GHJ dislocation or subluxation.

#### Patient-reported outcomes

Included participants were asked to complete the Melbourne Instability Shoulder Score (MISS) and Western Ontario Shoulder Index (WOSI) questionnaires preoperatively and between 12 and 24 months postoperatively.^19^^,^^37^ The MISS is a self-administered tool, with a total of 100 points, divided into 4 categories that assess pain, instability, function, and occupational and sporting demands. The total score for the MISS can range between 0 and 100 points, where 100 represents no deficit. The minimally clinically important difference for the MISS is 5 points.^37^ The WOSI is a self-administered tool with 21 items over the 4 domains of physical symptoms, sport/recreation/work, lifestyle function, and emotional function. The total score is expressed as percentage, with 100% representing a normal, healthy shoulder. The minimally clinically important difference for the WOSI is 10.4 points.^19^ The MISS^37^ and the WOSI^19^ are valid, reliable, and sensitive tools for measuring changes in the shoulder instability population.^30^

### Statistical analyses

No *a priori* sample size calculation was undertaken as all patients meeting the study entrance criteria were enrolled. A *post hoc* power analysis indicated that the study had a power of 0.32 to demonstrate a significant difference in the observed bony union rates of the 2 groups.

Some patients who underwent a unicortical fixation had small glenoid fossae, and the bone screw traversed both cortices. However, we undertook “intention to treat” analyses, and these patients remained in the unicortical group.

For rate of bony union, recurrence of instability, and postoperative complications, results were reported descriptively using point estimates and levels of uncertainty: median (interquartile range) and percentages (95% confidence intervals [95% CI]). Categorical data were analyzed using the Chi-squared and Fisher’s exact tests and differences in proportion (95% CI). The continuous data were nonparametric and analyzed using the Mann-Whitney U test.

Completed sets of preoperative and postoperative PROMs were available for 30 participants in the unicortical group and only for 4 participants in the bicortical group. The online system developed to collect PROMs in the surgeon’s practice was only implemented in late 2017, and therefore, patients operated on prior to this (ie, the majority of the bicortical group) did not have baseline PROMs for postoperative comparison. Due to the very low sample of PROMs in the bicortical group, between-group statistical analysis (unicortical vs. bicortical) and within-group analysis for the bicortical group only could not be performed as it would be significantly underpowered. As PROMs data were nonparametric, within-group analysis for the unicortical group was performed using related samples Wilcoxon signed-rank tests.^41^ Descriptive statistics (means, medians, median differences, and percentages) were reported for both groups.

All data were analyzed with SPSS for Windows statistical software (SPSS version 26.0; IBM Corp., Armonk, NY, USA). The level of significance was 0.05. All authors had access to the raw data to reduce potential sources of bias.

## Results

### Patient characteristics

**Figure 1:**
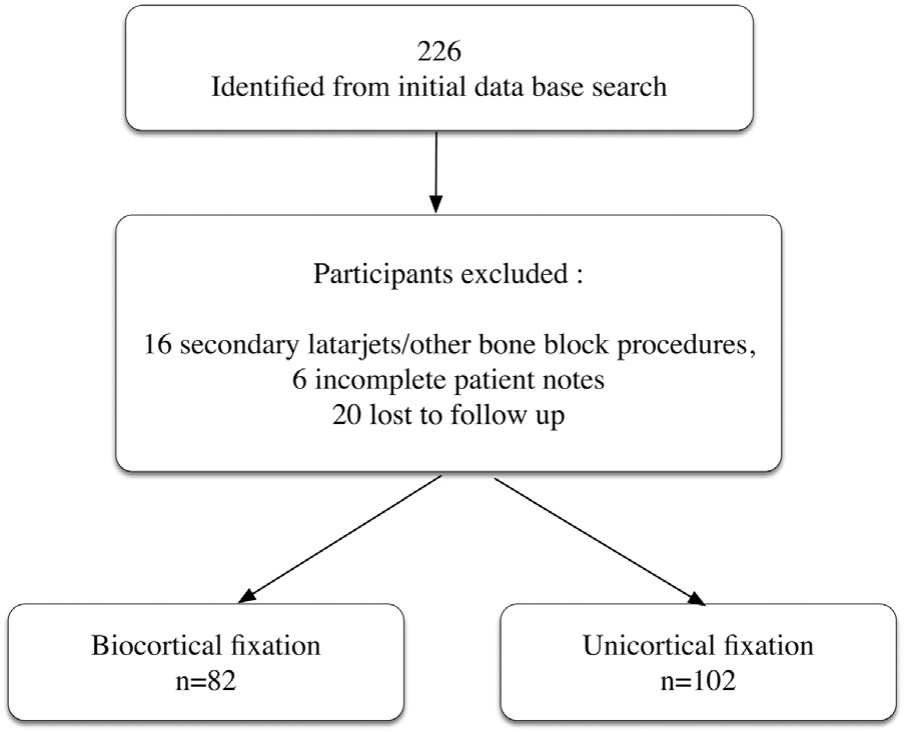
Flow chart of inclusion of participants in the study.

A total of 226 patients were identified from the database for potential inclusion into the study. Patients were excluded for the following reasons: Sixteen were secondary Latarjets or had other bone block procedures, 6 for incomplete patient notes, and 20 were lost to follow-up. A total of 184 participants were included in the study with surgery dates ranging from March 2015 to August 2019. Of this total, 82 participants had bicortical screw fixation, and 102 patients had unicortical screw fixation of their graft (Fig. 1).

There was no difference in baseline characteristics between participants enrolled in the study and those lost to follow-up (Table I). The baseline characteristics of the participant groups are outlined in Table II. The groups did not differ in proportion of gender, age at the time of surgery, side of the operation, nature of imaging undertaken, time to postoperative imaging, nature of the operation (primary or revision procedure), or additional procedures required (*P* > .05).

**Table I:** Summary comparison of participants enrolled in the study and those lost to follow-up.

Characteristic, n (%)	Patient status		
	Enrolled, N = 184	Lost to follow-up, N = 20	Difference in proportions, % (95% CI)
Unicortex	102 (55.4)	12 (60.0)	4.6 (−20.8, 30.0)
Male gender	165 (89.7)	20 (100)	10.3 (−3.1, 17.55)
Right shoulder operated upon	94 (51.1)	9 (45.0)	6.1 (−19.6, 31.8)
Patients' primary operation	129 (70.1)	15 (75.0)	4.9 (−18.0, 27.8)
Additional procedure required	6 (3.3)	2 (10.0)	6.7 (−9.4, 22.9)
Operative complication occurred	9 (4.9)	0 (0.0)	4.9 (−1.0, 10.8)

*CI*, confidence interval.

**Table II:** Baseline characteristics and outcomes between bicortical and unicortical fixation.

Characteristics	Bicortical, n = 82	Unicortical, n = 102	*P* value
Gender, n (%)			
Male	75 (91.5)	90 (88.2)	.47
Female	7 (8.5)	12 (11.8)	
Side of operation, n (%)			
Right	40 (48.8)	54 (52.9)	.57
Left	42 (51.2)	48 (47.1)	
Imaging, n (%)			
CT scan	20 (26.8)	28 (27.5)	.93
X-ray	60 (73.2)	74 (72.5)	
Nature of the operation, n (%)			
Primary operation	56 (68.3)	73 (71.6)	.63
Not primary operation	26 (31.7)	29 (28.4)	
Additional procedure status, n (%)			
Required	3 (3.7)	3 (2.9)	.79
Not required	79 (96.3)	99 (97.1)	
Age at the time of operation, yr, median (IQR)	23.1 (7.1)	24.6 (10.6)	.45
Time to imaging after operation, weeks, median (IQR)	10.0 (5.0)	9.6 (4.6)	.11
Outcomes			
Full graft union, n (%)	80 (94)	100 (98)	.25
Postoperative complication, n (%)			
Present	5 (6.1)	4 (3.9)	.50
Not present	77 (93.9)	98 (96.1)	
Recurrence, n (%)			
Occurred	2 (2.4)	2 (2.0)	.83
Did not occur	80 (97.6)	100 (98.0)	

*CT*, computed tomography; *IQR*, interquartile range.

In the bicortical group, 56 of the 82 cases were primary Latarjet procedures, and the remaining 26 were revision procedures (23 from single arthroscopic Bankart repair, 1 from multiple arthroscopic Bankart repairs, and 2 from open Bankart repairs). In addition to the Latarjet, 3 patients had an open humeral avulsion glenohumeral ligament repair, and 1 had an arthroscopic posterior capsule reconstruction. In the unicortical group, 73 cases of the 102 participants were primary Latarjet procedures, and the remaining 28 were revision procedures (22 from single arthroscopic Bankart repair, 2 from multiple arthroscopic Bankart repairs, and 4 from open Bankart). In the unicortical group, 9 patients were found to have bicortical graft fixation with both screws on postoperative imaging, with 2 others having 1 screw unicortical and the second screw bicortical. In addition to the Latarjet, 1 patient had an open humeral avulsion glenohumeral ligament repair, and 2 had an arthroscopic posterior capsule reconstruction.

### Radiographic

**Figure 2:**
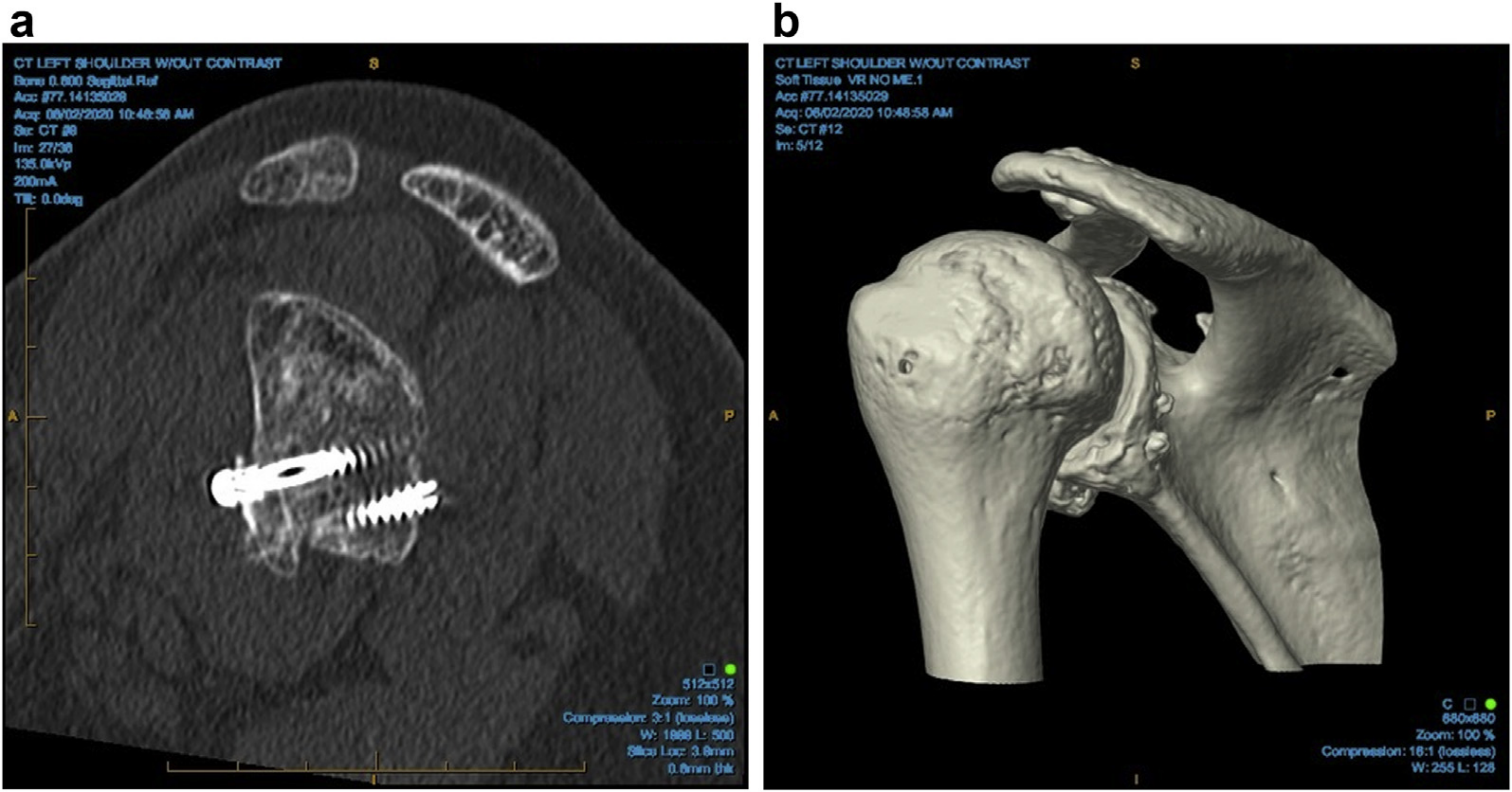
Bicortical screw fixation on (**a**) CT and (**b**) 4D CT scan. *CT*, computed tomography.

**Figure 3:**
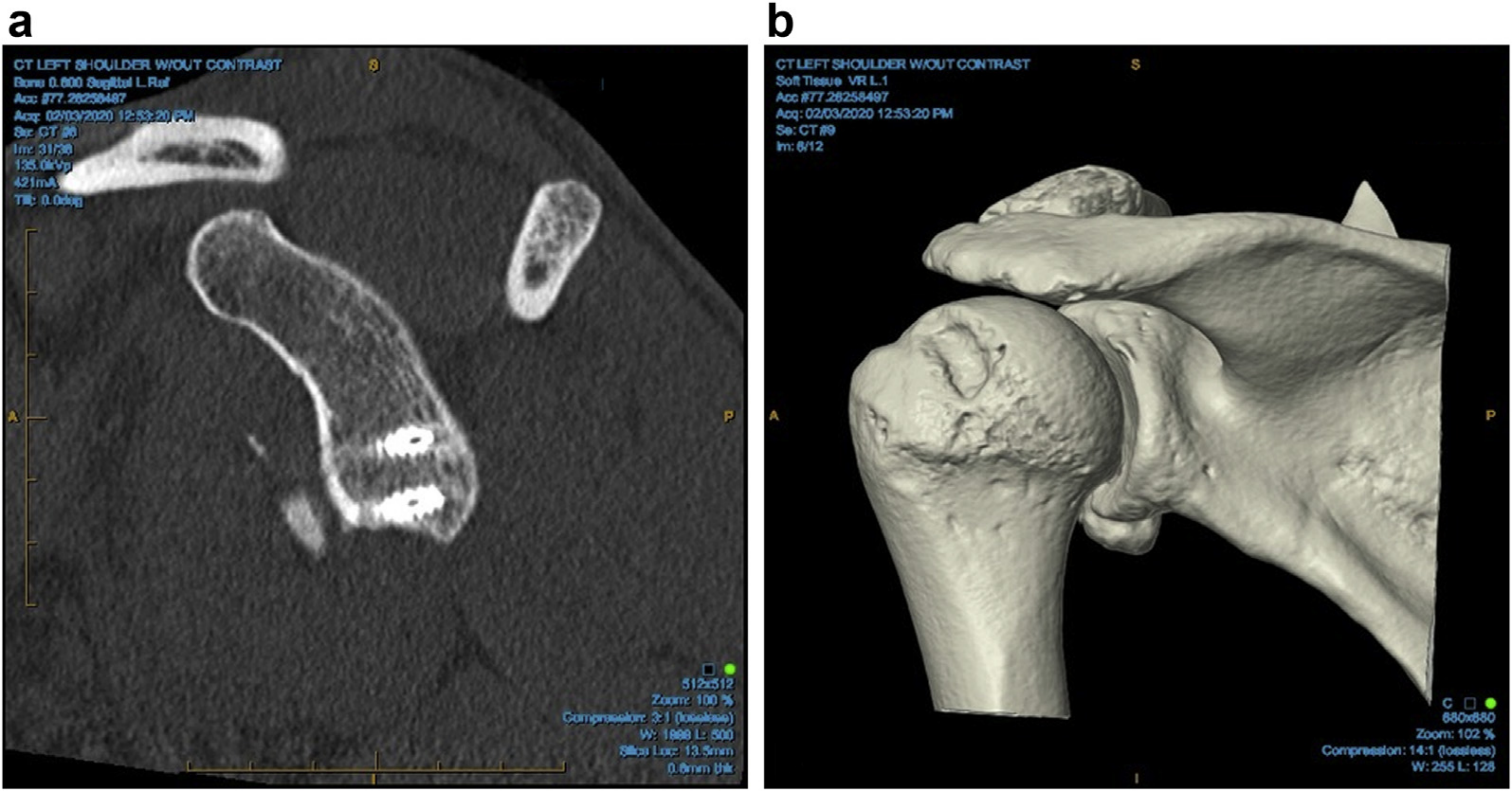
Unicortical screw fixation on (**a**) CT and (**b**) 4D CT scan. *CT*, computed tomography.

In the bicortical group, 80 of the cases (94%) went on to full graft union. Sixty of these were seen on plain x-rays at 8 weeks, and 20 were seen on subsequent CT scans (Fig. 2, *A* and *B*) at a mean of 14.1 weeks (range 8-25 weeks). In the unicortical group, 100 cases (98%) went on to full graft union. Of these, 74 were seen on plain x-rays at 8 weeks, and 26 were seen on subsequent CT scans (Fig. 3, *A* and *B*) at a mean of 10.8 weeks (range 8-28 weeks). The 2 remaining cases form the unicortical group, as well as 2 nonunion cases from the bicortical group, had radiographic evidence of significant bony resorption but no symptoms of pain or instability and have not had any recurrences.

Between-group comparison for bony union revealed that 100 (98.0%) unicortical and 80 (94%) bicortical patients had union of their graft (difference in proportions: 4.1%, 95% CI: −2.8, 11.1, *P* = .25). There was no suggestion of a difference between a primary and revision operation influencing the rate of nonunion, with 3.9% (5 of 129 patients) of primary operation patients having a nonunion, compared with 3.6% (2 of 55 patients) of revision-operation patients.

### Recurrence of instability

There was no significant difference between groups for recurrence of instability (difference in proportions: 0.4%, 95% CI: −4.9, 5.9, *P* = .83). In the bicortical group, 2 (2.4%) patients had a recurrence of instability. One of these patients had imaging that showed a nonunion associated with screw backout. The patient underwent a revision to an Eden-Hybinette procedure with iliac crest graft, and at the last follow-up, the patient was asymptomatic. This case of recurrence was the only 1 in our cohort associated with nonunion. The other patient had a single dislocation episode but did not elect for further surgery and has not represented with any further episodes of instability. In the unicortical group, 2 (2%) patients had a recurrence of instability. The first was managed nonoperatively and has since had no further episodes of instability. The second was diagnosed as having multidirectional instability and required a subsequent arthroscopic posterior capsular reconstruction. At the last follow-up, the symptoms had resolved.

### Postoperative complications

There was no significant difference between groups for postoperative complications (difference in proportions: 2.2%, 95% CI: −5.3, 9.7, *P* = .50). In the bicortical group, 5 (6.1%) patients experienced a postoperative complication. One patient had an infection requiring washout and antibiotics and later a hydrodilatation for GHJ stiffness. At the last follow-up, the symptoms had settled. Two patients had a partial wound dehiscence that settled with antibiotics. One patient had persistent stiffness that improved following a hydrodilatation. One patient had a transient axillary nerve palsy that made a full recovery. In the unicortical group, 4 (3.9%) patients experienced a postoperative complication. Three patients had persistent stiffness that improved following a hydrodilatation. Another patient had an implant fracture following trauma and required another revision. No complication cases were associated with nonunion.

### Patient-reported outcome measures

PROM results are detailed in the online Supplementary Appendix S1. The mean follow-up time was 510 days (12-24 months). For the unicortical group, within-group analysis revealed a significant improvement postoperatively for all subsections (pain, instability, function, occupation, and sport) and total score of the MISS (median of the difference between baseline and follow-up MISS total score: 29.5 points, 95% CI: 22.5-35.5, *P* < .001), as well as for all subsections (physical symptoms, sport, lifestyle, emotion) and total score for the WOSI (median of the difference between baseline and follow-up WOSI total score: 36.7 points, 95% CI: 28.8-46.2, *P* = .000). For the bicortical group, descriptive reporting revealed an improvement in all subsections and total score of the MISS (median of the difference between baseline and follow-up MISS total score: 28.5 points, 95% CI: 13-47) and all subsections and total score of the WOSI (median of the difference between baseline and follow-up WOSI total score: 31.9 points, 95% CI: 21.1-61.2).

## Discussion

The benefits of the Latarjet procedure have been well documented, with its important role in providing GHJ stability in patients with significant bone loss, failed primary procedures, and in athletes mandating return to elite contact sport.^3^^,^^7^^,^^10^^,^^27^^,^^29^ In this study, there was no significant difference in the rate of bony union between participants undergoing a Latarjet with unicortical vs. those with bicortical screw fixation. However, the absolute numbers of patients with nonunion were small, and the study was underpowered to demonstrate a statistically significant difference. To our knowledge, there have been no similar studies in the literature explicitly comparing outcomes for bicortical vs. unicortical fixation, and therefore, this study is the first of its kind.

In our unicortical fixation group, we did not see a significant graft dissociation rate due to nonunion compared to our bicortical group, despite *in vitro* studies suggesting an inferior graft pull-out strength of the former.^2^^,^^34^ We had no episodes of patients complaining of pain secondary to prominent posterior glenoid metalwork. Furthermore, we did not see a difference in outcomes (PROMS) or recurrence based on graft union contrary to studies suggesting that bony union has correlation with clinical outcomes.^24^ This could be explained by the fact that the benefit of the Latarjet is multimodal, as alluded to previously, with stability provided not only by the bony graft but also with a contribution from the capsular repair and the sling effect of the conjoint tendon.

The small nonunion numbers (with lack of statistical significance) mean we cannot claim unicortical fixation as a better method. However, our results support our hypothesis that the unicortical fixation did not result in an inferior union rate. In addition, there was no suggestion of a difference between a primary and revision operation influencing the rate of nonunion, with 3.9% of primary operation patients having a nonunion compared with 3.6% of revision operation patients. Furthermore, nonunion did not appear to be associated with recurrence or other complications as only 1 case with nonunion had a postoperative instability episode; however, this relationship is speculative as the sample with nonunion was too small to perform a correlation analysis.

Interestingly, 2 patients from each group had evidence of bone resorption on imaging. All patients were asymptomatic with no reports of recurrence of instability. This finding is similar to that in previous studies^43^^,^^44^ that have reported high rates of bony resorption on imaging after a Latarjet procedure with no effect on recurrence rates or clinical outcomes. However, future studies correlating bony resorption with clinical outcomes after a Latarjet procedure should use instability-specific outcome measures to determine the effect of bony resorption more accurately on a patients’ instability symptoms.

For PROMs, the unicortical group showed a statically significant improvement postoperatively for the total MISS and WOSI scores, as well as all their related subsections. The median of the difference between baseline and follow-up for the total score of the MISS and the WOSI exceeded the minimal clinical important difference (MCID MISS = 5 points, MCID WOSI = 10.4%) indicating not only a statistical difference but also a clinically important improvement postoperatively. The significant improvement in the subsection of function (MISS) and sport (MISS and WOSI) suggests that return to activity and sporting participation had increased for participants after surgery; however, it is unknown to what degree compared to preinjury levels. Due to the very low numbers in the bicortical group, a between-group analysis or within-group analysis for this group could not be performed. However, the estimated median differences postoperatively for the total MISS and WOSI scores for the bicortical group also exceeded the MCID, indicating a clinically important improvement. The generalizability of the bicortical group’s results to a larger sample of patients undergoing a Latarjet procedure with unicortical screw fixation must be interpreted with caution due to the small sample size.

There are numerous reports of complications associated with bicortical screw fixation, which may make the use of unicortical fixation more appealing. Plessis et al,^31^ in a cohort of 192 patients, reported a suprascapular nerve injury and 5 other screw-related complications (including prominent screws and bent screws) following the Latarjet procedure with bicortical fixation. In a 90-day follow-up study, Frank et al^6^ reported 10 complications in their cohort of 156 bicortical Latarjet procedures, including a musculocutaneous nerve injury. In 2 separate studies, Maquieria et al^25^ and Sastre et al^33^ report on a case of suprascapular nerve injury due to insult with the superior screw of the bicortical fixation. Some experts believe that the incidence of suprascapular nerve injury after bicortical fixation is in fact underreported.^12^ Although these bicortical related complications are low overall, they should be considered in the context of clinical decision-making. In this study, there was no difference in complications between unicortical and bicortical fixation; however, a larger sample may have enabled detection of between-group differences. Given the lack of difference in union rates between the unicortical and bicortical groups in this study, it would be considered an acceptable practise to use unicortical fixation.

The limitations in our study are a lack of randomization and being underpowered to demonstrate an observed difference in bony union between the 2 groups. Assessing nonunion using plain radiography is challenging, and we acknowledge that CT may be superior for assessing graft union; however, we believe that judicious use of CT scanning in cases where there is clinical or radiological uncertainty is preferable to routine CT scanning due to the radiation involved. In addition, although there were no statistically significant differences between the patients enrolled and those lost to follow-up, there were more postoperative complications in the patients enrolled. This may have introduced selection bias. There may have been an effect of unicortical fixation patients effectively receiving bicortical fixation by virtue of their anatomy. As all patients were operated on by a single surgeon, these findings may lack external validity. Another limitation is the small proportion of PROMs that were available, particularly for the bicortical group. This is primarily due to the fact that the majority of patients in the bicortical group had their operation prior to 2018, preceding the implementation of the online database for the collection of PROMs. Despite these small numbers, PROMS are an important component of measuring patient-perceived disease-specific health and well-being^26^ and provide a preliminary indication of patient-reported success. The follow-up interval was relatively short (12-24 months); however, the primary outcome of interest was union rate, which is determined over short-term follow-up. Furthermore, complications from the Latarjet typically occur within 1 year, except for instability recurrence, which has a higher risk profile after Bankart repair.^29^ Future studies should investigate screw fixation type in relation to specific return to sport questions, recurrence, and instability-specific PROMS with long-term follow-up.

## Conclusion

This study revealed that there was no clinically significant difference in union rates between the unicortical and bicortical coracoid fixation methods for patients undergoing the Latarjet procedure. The number of patients with nonunion in both groups were small, and the study was underpowered to demonstrate a statistically significant difference. This study suggests that continuing to perform unicortical fixation is an acceptable practice as it does not compromise on graft union.

## Acknowledgment

The authors would like to acknowledge Imaging@ Olympic Park, Melbourne, for facilitating image retrieval for patients.

## Disclaimers:

Funding: Johnson & Johnson and Arthrex provide funds for fellowship programs that contribute to the completion of this research. There is no grant number for this fellowship funding. Johnson & Johnson nor Arthrex were involved in the development, data collection, analysis, or writing of this manuscript.

Conflicts of interest: Shivan S. Jassim received fellowship sponsorship while participating in this study (Johnson & Johnson/Arthrex). Jeevaka Amaranath received fellowship sponsorship while participating in this study (Johnson & Johnson/Arthrex). Sarah Ann Warby is employed as a research coordinator on a casual basis by Gregory Hoy. Sarah Ann Warby’s casual employment has contributed to the writing of this paper. Gregory Hoy is a consultant for Johnson & Johnson who make the screw for both types of fixation methods used in this study. The other author, his immediate family, and any research foundation with which he is affiliated have not received any financial payments or other benefits from any commercial entity related to the subject of this article.

## Supplementary Data

Supplementary Appendix S1

STROBE_checklist
