# Supplementary material for: Unicortical fixation does not compromise bony union in the Latarjet procedure
Source: JSES Int. 2022 May 13;6(4):555–62. doi: 10.1016/j.jseint.2022.04.007 (PMC9264013; doi:10.1016/j.jseint.2022.04.007)
Supplement: Supplementary Appendix S1 [file mmc1.docx]

Patient Reported Outcomes

| Outcome | N | Mean (SD) | | Median | | Median Difference  (95 % CI) | Significance |
| --- | --- | --- | --- | --- | --- | --- | --- |
|  |  | **Pre** | **FU^** | **Pre** | **FU^** | (Within-group) | |
| **MISS TOTAL** | *Score from 0 to 100% with a higher percentage representing a higher functioning shoulder* | | | | | | |
| *Unicortical* | 30 | 51.1 (16) | 80.1 (11.7) | 48.5 | 80 | 29.5 (22.5 to 35.5) | **0.000** |
| *Bicortical* | 4 | 40.3 (14.3) | 68.8 (18.1) | 39.5 | 76 | 28.5 (13 to 47) |  |
| *MISS Pain* | *Score from 0 to 15 points; a higher score indicates a lower level of pain* | | | | | | |
| *Unicortical* | 30 | 11.1 (3.1) | 12.3 (4.2) | 11 | 14 | 2 (0.5 to 3) | **0.017** |
| *Bicortical* | 4 | 9.5 (3) | 12.3(3.1) | 11 | 13 | 3.3 (-3 to 7) |  |
| *MISS Instability* | *Score from 0 to 33 points; a higher score indicates fewer instability symptoms* | | | | | | |
| *Unicortical* | 30 | 16.3 (7.4) | 26.7 (5.0) | 17 | 27 | 10.5 (7.5 to 14) | **0.000** |
| *Bicortical* | 4 | 10.8 (6.2) | 25 (5.4) | 10 | 23 | 14.3 (4 to 24) |  |
| *MISS Function* | *Score from 0 to 32 points; a higher score indicates a higher level of general shoulder function* | | | | | | |
| *Unicortical* | 30 | 14.4 (6.3) | 26 (6.3) | 15 | 28 | 12 (9 to 15) | **0.000** |
| *Bicortical* | 4 | 14.0 (5.0) | 19.3 (8.8) | 14.5 | 20.5 | 5.5 (0 to 9) |  |
| *MISS Sport/Occup.* | *Score from 0 to 20 points; a higher score indicates a higher level of sporting/occupational function* | | | | | | |
| *Unicortical* | 30 | 9.3 (2.9) | 15 (2.7) | 9 | 15 | 6 (5 to 7) | **0.000** |
| *Bicortical* | 4 | 6.0 (4.1) | 12.3 (5.6) | 6 | 12.5 | 7.3 (-1 to 11) |  |
| **WOSI TOTAL** | *Score from 0% to 100%, 100% = a normal shoulder; a higher percentage indicates a higher-functioning shoulder* | | | | | | |
| *Unicortical* | 29 | 40.9 (35.9) | 76.8 (17.7) | 35.9 | 83.4 | 36.7 (28.8 to 46.2) | **0.000** |
| *Bicortical* | 4 | 29.5 (17) | 65.2 (20) | 276.5 | 62.9 | 31.9 (21.1 to 61.2) |  |
| *WOSI physical* | *Score from 0 to 100 points; a lower score indicates a lower level of shoulder physical disability* | | | | | | |
| *Unicortical* | 29 | 527.3 (196.6) | 234.5(191.2) | 574.0 | 150. | -307 (-396 to -208) | **0.000** |
| *Bicortical* | 4 | 632.5(153.4) | 278.8 (149.7) | 686.0 | 271.0 | -353.8 (-467 to -219) |  |
| *WOSI Sport* | *Score from 0 to 40 points; a lower score indicates a lower level of shoulder sporting disability* | | | | | | |
| *Unicortical* | 29 | 265.1 (82.4) | 87.5 (74.1 | 275.0 | 77.0 | -176 (-212 to -143.5) | **0.000** |
| *Bicortical* | 4 | 286.8 (82.8) | 138.3 (192.2) | 292 | 126.5 | -130.3 (-348 to -43) |  |
| *WOSI Lifestyle* | *Score from 0 to 40 points; a lower score indicates a lower level of shoulder lifestyle disability* | | | | | | |
| *Unicortical* | 29 | 223.4 (78.7) | 79.1 (78.5) | 240.0 | 63 | -48.5 (-186.5 to 111.5) | **0.000** |
| *Bicortical* | 4 | 308.3 (77.6) | 176.3(95.7) | 315.5 | 143 | -111(-246 to -74) |  |
| *WOSI Emotion* | *Score from 0 to 30 points; a lower score indicates a higher level of shoulder emotional disability* | | | | | | |
| *Unicortical* | 29 | 225.5 (68.6) | 85.5 (76.6) | 245.0 | 78 | -145 (-180 to -108) | **0.000** |
| *Bicortical* | 4 | 253.0 (65.6) | 137 (119.3) | 276.5 | 133.5 | -116 (-224 to -27) |  |

*Note.* Significance level set at p<0.05. ^ Mean follow up time 509 days (12 to 24 months). Values reaching significance are highlighted in bold. MISS= Melbourne Instability Shoulder Score, WOSI= Western Ontario Shoulder Index, FU= follow up. MISS TOTAL = 0 to 100 with 100 representing a normal functioning shoulder. WOSI TOTAL= 0 to 100 with 100 representing a normal functioning shoulder. Minimal Clinical Important Difference MISS (TOTAL)= 5 points. Minimal Clinical Important Difference WOSI (TOTAL) =10.4 points
